# Boosted Electrocatalytic Degradation of Levofloxacin by Chloride Ions: Performances Evaluation and Mechanism Insight with Different Anodes

**DOI:** 10.3390/molecules29030662

**Published:** 2024-01-31

**Authors:** Keda Yang, Peiwei Han, Yinan Liu, Hongxia Lv, Xiaofei Chen, Yihan Lei, Lian Yu, Lei Ma, Pingzhou Duan

**Affiliations:** 1 Shulan International Medical College, Zhejiang Shuren University, Hangzhou 310015, China; kdyang@zjsru.edu.cn; 2 Beijing Key Laboratory of Fuels Cleaning and Advanced Catalytic Emission Reduction Technology, College of New Materials and Chemical Engineering, Beijing Institute of Petrochemical Technology, Beijing 102617, China; hanpeiwei025@163.com (P.H.); bipt_liuyinan@163.com (Y.L.); 2019520036@bipt.edu.cn (H.L.); 3 Chen Ping Laboratory of TIANS Engineering Technology Group Co., Ltd., Shijiazhuang 050000, China; chenxiaofei@tianjushi.com (X.C.); leiyihan@tianjushi.com (Y.L.); 4 Department of Environmental Engineering, Beijing Institute of Petrochemical Technology, Beijing 102617, China; yulian@bipt.edu.cn; 5 State Key Laboratory of Environmental Criteria and Risk Assessment, Chinese Research Academy of Environmental Sciences, Beijing 100012, China

**Keywords:** electrocatalytic oxidation, levofloxacin wastewater, degradation efficiency, chloride ion, reaction mechanism

## Abstract

As chloride (Cl^−^) is a commonly found anion in natural water, it has a significant impact on electrocatalytic oxidation processes; yet, the mechanism of radical transformation on different types of anodes remains unexplored. Therefore, this study aims to investigate the influence of chlorine-containing environments on the electrocatalytic degradation performance of levofloxacin using BDD, Ti_4_O_7_, and Ru-Ti electrodes. The comparative analysis of the electrode performance demonstrated that the presence of Cl^−^ improved the removal and mineralization efficiency of levofloxacin on all the electrodes. The enhancement was the most pronounced on the Ti_4_O_7_ electrode and the least significant on the Ru-Ti electrode. The evaluation experiments and EPR characterization revealed that the increased generation of hydroxyl radicals and active chlorine played a major role in the degradation process, particularly on the Ti_4_O_7_ anode. The electrochemical performance tests indicated that the concentration of Cl^−^ affected the oxygen evolution potentials of the electrode and consequently influenced the formation of hydroxyl radicals. This study elucidates the mechanism of Cl^−^ participation in the electrocatalytic degradation of chlorine-containing organic wastewater. Therefore, the highly chlorine-resistant electrocatalytic anode materials hold great potential for the promotion of the practical application of the electrocatalytic treatment of antibiotic wastewater.

## 1. Introduction

Since the 1990s, with the rapid advancement of national urbanization and the industrial level in China, the overuse of levofloxacin as an anti-inflammatory agent for humans has been highlighted as a critical environmental threat [1]. Notably, levofloxacin is physio-chemically stable with a prolonged half-life, which results in its inevitable entrance into the water environment, where it causes irreversible harm to human beings, such as acute renal failure, vomiting, allergies, etc. [2]. In updated studies, the primary sources of levofloxacin were discovered in wastewater discharged from healthcare facilities and pharmaceutical manufacturing, groundwater, and even drinking water [3,4]. Consequently, advanced water treatment technologies with green safety, efficient catalysis, and high equipment flexibility are eagerly sought to minimize the ecological risk of levofloxacin contamination in the environment.

The conventional methods for treating antibiotics, such as the biochemical method [5,6,7,8], adsorption method [9,10], and membrane separation method [11,12], have various disadvantages, such as low removal efficiency, easily caused toxic accumulation, and secondary pollution of the terrestrial ecosystem. At present, the advanced oxidation processes, such as ozone oxidation [13], Fenton oxidation [14,15], catalytic wet oxidation [16,17], and photocatalytic oxidation [18,19], are limited in their wide application due to their selective oxidation capacity, the long reaction time required, harsh conditions, high cost, and low application scope. Electrocatalytic oxidation, which is one of the advanced oxidation processes, has been widely considered by scholars due to its advantages in terms of simple operation, high degradation efficiency, and high mineralization efficiency. Central to electrochemical oxidation processes are the anode materials, which mainly contain the boron-doped diamond (BDD) film electrode, titanium electrode [20,21,22,23], and metal oxide electrode [24,25]. Based on the intrinsic nature of anodes, both the nonradical and the radical oxidation mechanisms were illustrated in the contributions to the degradation of antibiotics by the synergistic effects of direct electrolysis and indirect oxidations. Notably, the in situ generation of •OH on the non-active anode was regarded as the dominant pathway for organic degradation [26,27,28,29].

As an extremely ubiquitous anion, the chloride ion performed an important role in the degradation mechanism of the electrode used in the electrocatalytic oxidation processes [28,30,31,32,33,34]. Recent studies suggest that the oxidizing agents (H_2_O_2_, O_3_, and PDS) in sulfate and chloride electrolyte media resulted in different electrolysis performances [35,36]. As an equally important free radical agent, it was found that the influence of co-existing anions on SO_4_^•−^ in the degradation of atrazine followed the order of chloride > sulfate > bicarbonate > dihydrogen phosphate > nitrate ions [37,38,39]. It also indicated that chloride ions boosted the electrochemical oxidation of perfluorooctanesulfonate by Ti_4_O_7_ and BDD anodes [40,41]. However, the respective contribution of electrolysis and the different radicals which are responsible for the removal of pollutants on different anode materials has not yet been reported.

Although there are numerous studies on the electrocatalytic oxidation in chloride conditions, the comprehensive insight into and quantitative evaluation of the degradation mechanism of levofloxacin by the BDD, Ti_4_O_7_, and DSA system in chloride electrolyte media should be advanced further. In this paper, the BDD electrode, Ti_4_O_7_ electrode, and ruthenium–titanium electrode were selected as electrode materials to explore the degradation effect of the chloride ion content on levofloxacin and the degradation mechanisms of the electrodes. Firstly, the effects of NaCl concentration and reaction time on the performance of the electrodes were investigated using the total organic carbon (TOC) removal rate as the key technical index. Secondly, the morphology and properties of the electrodes were analyzed by means of XRD, SEM, and LSV and by other characterization means, and the changing law of radicals in the reaction was monitored by EPR characterization to explore the reaction mechanism. This study reveals the mechanism of chlorine ion participation in the electrocatalytic degradation of chlorine-containing organic wastewater. In addition, this study provides a theoretical reference for the development of catalysts for the efficient treatment of salt-containing organic wastewater.

## 2. Results and Discussion

### 2.1. Characterization of Catalytic Electrodes

**Figure 1:**
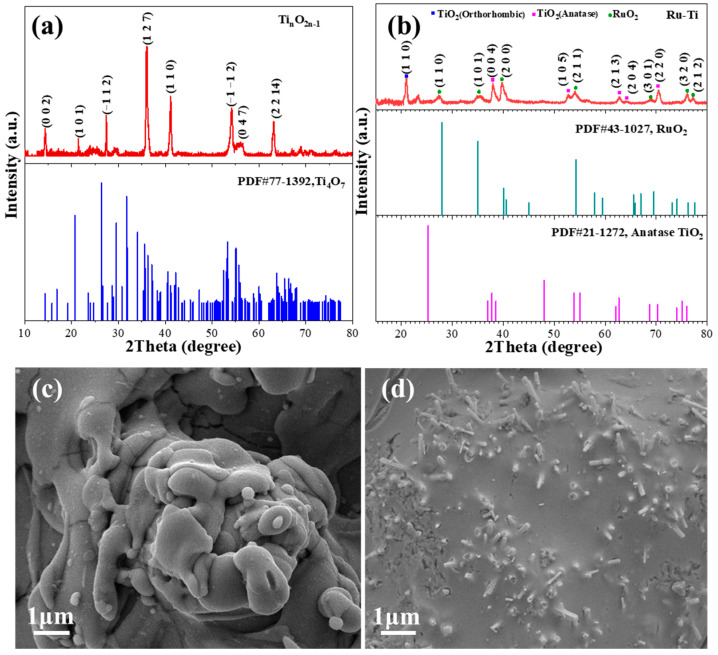
XRD patterns of (**a**) Ti_n_O_2n−1_, (**b**) Ru-Ti; SEM images of (**c**) Ti_n_O_2n−1_, (**d**) Ru-Ti.

The crystal structures of the three anode materials were characterized by X-ray diffraction (XRD). As shown in Figure S2a, the synthesized BDD material is consistent with the standard characteristic spectrum (PDF#06-0675) [42,43]. The characteristic diffraction peaks at 43.9 and 75.3 of the BDD anode corresponded to the (111) and (220) crystal planes, respectively. Figure 1a shows the surface crystalline phase structure of the titanium suboxide anode, and it can be clearly observed that the characteristic peaks correspond to the standard characteristic mapping of the triclinic phase of Ti_4_O_7_ (PDF#77-1392) [44,45]. The characteristic peaks at 14.3°, 21.4°, 27.4°, 36°, 41°, 54°, and 63.2° of the titanium suboxide anode could be assigned to the (002), (101), (−112), (127), (110), (121), and (149) crystal planes, respectively. The characteristic peaks of the Ru-Ti anode were matched with the RuO_2_ standard characteristic spectrum (PDF#43-1027) and the TiO_2_ standard characteristic spectrum (PDF#21-1272) (Figure 1b). Figure 1c,d show the surface micromorphology of the two electrodes. The SEM image of the BDD electrode shows that the surface consists of angular and disordered polycrystalline grains (Figure S2b). Figure 1c exhibits the uneven surface and the presence of the pronounced pore structure of the titanium suboxide electrode. It may be beneficial to increase the active surface area and thus promote surface electron transport. The presence of rod-like structures can be clearly observed on the surface of the Ru-Ti electrode (Figure 1d).

### 2.2. Electrochemical Properties of Catalytic Electrodes

**Figure 2:**
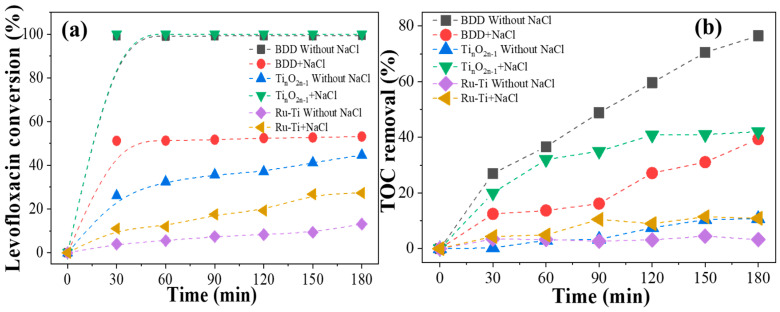
Degradation of levofloxacin by BDD electrode, titanium suboxide electrode, and Ru-Ti electrode in response to (**a**) LVX conversion rate and (**b**) TOC removal efficiency.

The electrocatalytic degradation performance of the electrodes was further explored by using different anodes, including BDD, titanium suboxide, and Ru-Ti electrodes for the degradation of levofloxacin. As shown in Figure 2a, which compares the degradation efficiency of the three electrodes, the BDD electrode performed the best, with cresol being completely degraded within 30 min. In contrast, the degradation efficiency of levofloxacin by the titanium suboxide electrode and the Ru-Ti electrode achieved only 26% and 4% within 30 min, respectively. Meanwhile, NaCl was added during the reaction to investigate the effect of chloride ions on the catalytic performance of the electrode. The results indicated that the addition of chloride ions inhibited the catalytic performance of the BDD electrode, and the degradation efficiency decreased to 50% within 30 min. The titanium suboxide electrode and the Ru-Ti electrode showed opposite results, where the titanium suboxide catalytic efficiency reached nearly 100% within 30 min. As shown in Figure 2b, the TOC removal efficiency exhibits the same trend as the levofloxacin degradation efficiency, indicating that the presence of chloride ions has a significant influence on the catalytic performance of the electrode.

### 2.3. Effects of Chloride Ions on Electrochemical Properties

In order to further explore the mechanism of chloride ions, the hydroxyl radicals in the catalytic reaction of the titanium oxide electrode and the Ru-Ti electrode were detected by electron paramagnetic resonance (EPR). As shown in Figure S3a,b, the intensity of the hydroxyl radical signals during the catalytic degradation of levofloxacin at the titanium suboxide electrode was significantly enhanced by the addition of NaCl. This may be attributed to the presence of chloride ions accelerating the generation of hydroxyl groups during the electrocatalytic reaction at the titanium suboxide electrode, while the chloride ions may electrolyze on the anode surface to form active chlorine (Equations (1)–(6)) [46]. The combined action of hydroxyl radicals and active chlorine accelerated the degradation and mineralization of levofloxacin. In contrast, the hydroxyl radical signal during the reaction was not obvious for the Ru-Ti electrode (Figure S3c). A slight enhancement of the hydroxyl radical signal was observed with the addition of NaCl (Figure S3d). Combined with the evaluation results, it is speculated that hydroxyl radicals play an important role in the reaction process. Tert-butanol (TBA) is often used as a hydroxyl radical (•OH) bursting agent (Figure S4). Therefore, tert-butanol was employed to verify the role of the hydroxyl radicals in the electrocatalytic reaction process, thus further elucidating the degradation mechanism [47]. The results showed that the electrocatalytic performance of all three electrodes decreased after the addition of TBA, with the degradation efficiency of the BDD electrode being only 2.3% within 30 min. This may be attributed to the fact that hydroxyl radicals play a major role in the electrocatalytic degradation of levofloxacin by the BDD electrode. However, the degradation efficiency of the titanium suboxide electrode decreased to 81% within 30 min, and part of the decrease may be attributed to the role of the hydroxyl radicals. The titanium suboxide also maintained a high catalytic performance, which may be attributed to the generation of active chlorine. In the water treatment process, pH plays an important role [48]. The effect of the pH value on the degradation of chlorine-containing levofloxacin wastewater by the titanium suboxide electrode was investigated by response surface methodology (Figure S5). The results showed that the removal efficiency of TOC was optimal when the pH value was in the neutral range [49]. This may be attributed to the fact that hydrogen peroxide is produced at the cathode under acidic or alkaline conditions; however, hydrogen peroxide readily bursts hydroxyl radicals, leading to a reduction in degradation efficiency [50,51].

(1) $${2Cl}^{-}+•OH+H^{+}\to {Cl}_{2}+H_{2}O$$

(2) $${Cl}_{2}+H_{2}O\to HClO+H^{+}+{Cl}^{-}$$

(3) HClO→Cl•+•OH

(4) $${ClO}^{-}+•OH\to ClO•+{OH}^{-}$$

(5) $$\mathrm{Organic}\;\mathrm{Pollutants}+•OH\to {CO}_{2}+H_{2}O+\mathrm{inorganic}\;\mathrm{ions}$$

(6) $${ClO}^{-}+Organic\;Pollutants\to {CO}_{2}+H_{2}O+{Cl}^{-}+intermediates$$

### 2.4. Effect of Different Chloride Concentrations on Electrochemical Properties

**Figure 3:**
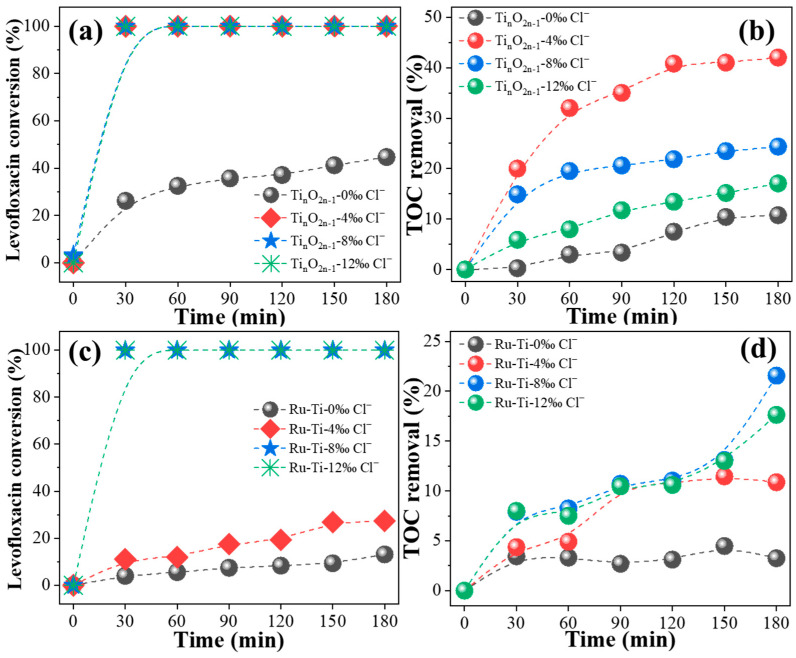
Effect of the amount of chloride ions on the conversion and TOC removal rate of levofloxacin degradation by titanium oxide electrode (**a**,**b**) and Ru-Ti electrode (**c**,**d**).

The effect of different concentrations of chloride ions on the performance of the electrocatalytic degradation of levofloxacin is shown in Figure 3. The results indicated that the electrocatalytic performance of the titanium suboxide electrode and the Ru-Ti electrode varied with the increase in chloride ion concentration. The addition of chloride ions improved the electrocatalytic performance of the titanium suboxide electrode. The degradation efficiency of levofloxacin reached about 100% within 30 min after the addition of chloride ions, while the TOC removal rate decreased with the increase in chloride ion concentration. In comparison, the best electrocatalytic performance of the titanium suboxide electrode was achieved with a 4‰ chloride addition. This may be attributed to the fact that at higher chloride ion concentrations, the active chlorine produced reacts with the pollutants to form chlorinated byproducts, resulting in reduced TOC removal. The Ru-Ti electrode showed a sharp increase in levofloxacin degradation efficiency to about 100% with 8‰ chloride ions, and it showed the best TOC removal performance. As with the titanium suboxide electrode, the TOC removal rate decreased with the increase in chloride ion concentration.

**Figure 4:**
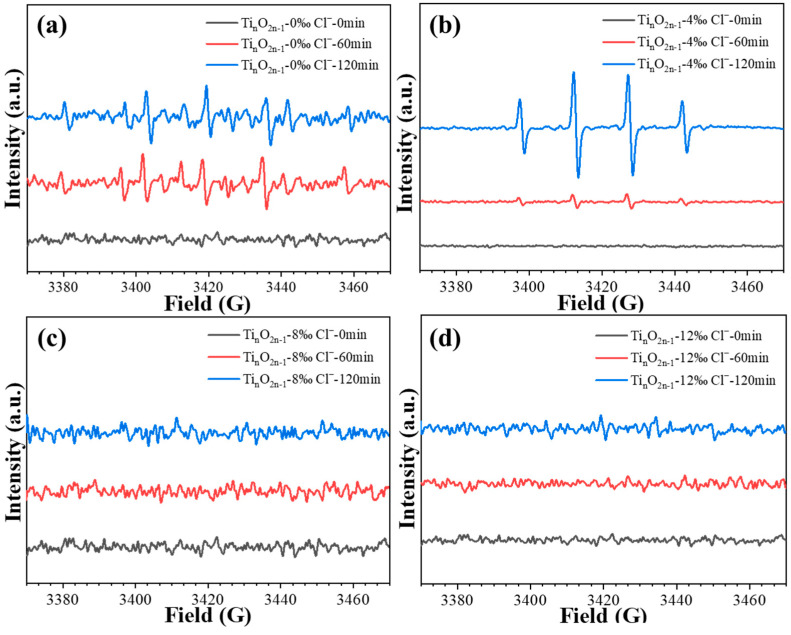
Effect of the amount of chloride ions on the EPR signal of hydroxyl radicals in the degradation of levofloxacin by titanium oxide electrode: (**a**) 0‰; (**b**) 4‰; (**c**) 8‰; (**d**) 12‰.

The effect of different concentrations of chloride ions on the concentration of hydroxyl radicals during the electrocatalytic degradation reaction of levofloxacin at the titanium suboxide electrode was further investigated. As shown in Figure 4, the signal intensity of the •OH radical showed an increasing and then decreasing trend with the increasing chloride ion concentration [52,53,54,55]. It can be observed that the amount of chloride ions is optimal at 4‰ of the hydroxyl radical signal strength, which corresponds to the evaluation results. With the continuing increase in the chloride ion concentration, the signal intensity of the hydroxyl radicals decreased. This may be attributed to the presence of excess chloride ions that can lead to the bursting of hydroxyl radicals [54,56]. Combined with the evaluation results, the decrease in the electrocatalytic performance of the titanium suboxide electrode may be related to the reduction in •OH radicals and the chlorinated byproducts.

### 2.5. Chlorine Free Radical Test

**Figure 5:**
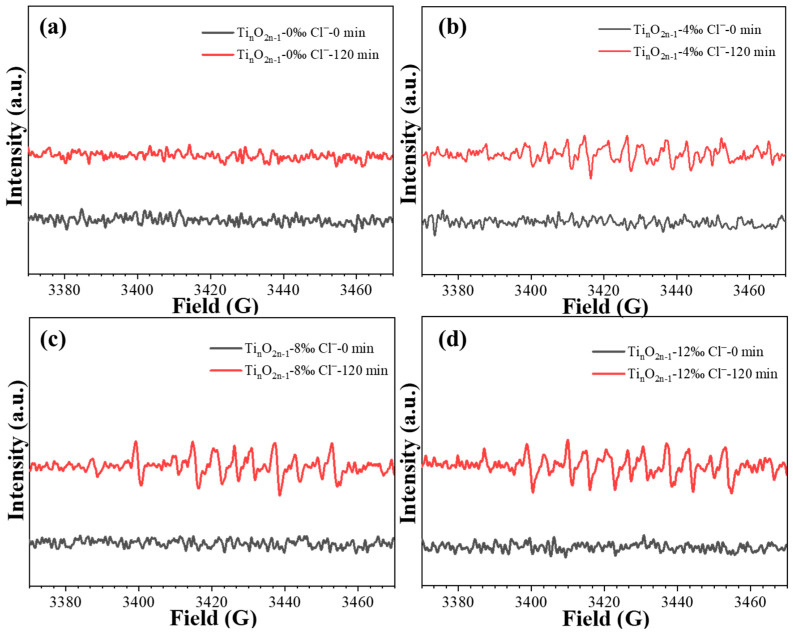
Effect of the amount of chloride ions on the EPR signal of chlorine radicals in the degradation of levofloxacin by titanium oxide electrode: (**a**) 0‰; (**b**) 4‰; (**c**) 8‰; (**d**) 12‰.

The results of the above analyses indicate that the generation of chlorine radicals has a great impact on the electrocatalytic efficiency of levofloxacin. DMPO was employed as the chlorine radical spin trapping agent to observe the generation of chlorine radicals during the reaction. As shown in Figure 5, the characteristic peak signals of the chlorine radicals were detected during the electrocatalytic reaction at the titanium suboxide electrode as the concentration of chloride ions increased [57]. In contrast, the chlorine radical characteristic peak signal intensity was optimal at a chloride ion concentration of 8‰. With the increase in the chloride ion concentration to 12‰, there was a slight decrease in the chlorine radical signal. This may be attributed to the depletion of chlorine radicals as they react with organic matter to form chlorinated byproducts. Combined with the evaluation results, keeping the chloride ion concentration in a suitable range can generate chlorine radicals that contribute to the degradation of levofloxacin during the reaction process at the titanium suboxide electrode [58,59]. The increased concentration of chlorine radicals leads to the formation of difficult-to-degrade chlorinated byproducts in a reaction with organic matter, which may be an important reason for the reduced efficiency of organic matter mineralization.

### 2.6. Electrochemical Testing

**Figure 6:**
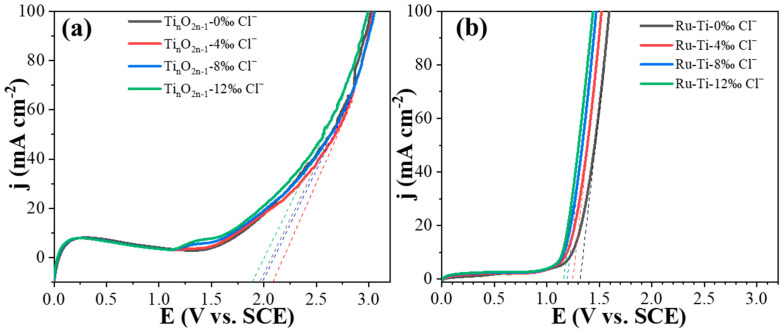
Electrochemical characterization–LSV curves of (**a**) the titanium suboxide electrodes and (**b**) Ru-Ti electrodes.

According to linear sweep voltammetry (LSV) characterization, the titanium suboxide electrode indicated excellent electrocatalytic properties for organic oxidation. The LSV characterization monitored the effect of different concentrations of chloride ions on the oxygen evolution potential (OEP) of the titanium suboxide electrodes and the Ru-Ti electrodes [60,61]. In correspondence with the above characterization results, the titanium suboxide electrode has the largest oxygen precipitation potential at a chloride ion concentration of 4‰ (Figure 6a, Table S1). This indicates that the oxygen generation barrier increases under this condition and thus promotes the generation of •OH radicals, which is also confirmed by the EPR characterization results. In contrast, the OEP of the Ru-Ti electrode gradually decreased with the increasing chloride ion concentration (Figure 6b). By combining the latter with the EPR characterization results, it was determined that this could be attributed to the presence of chloride ions occupying the surface of the Ru-Ti electrode, resulting in the hindrance of •OH radical generation and the consequent decrease in oxygen evolution potentials.

### 2.7. Exploration of Reaction Mechanism

**Figure 7:**
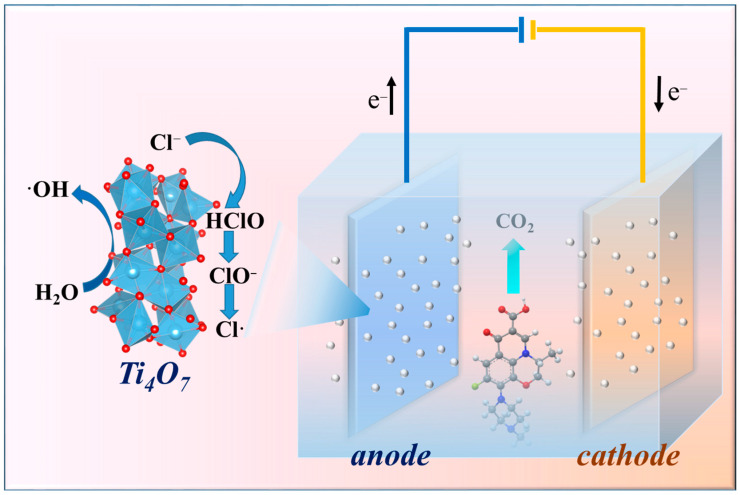
Mechanism of the electrochemical degradation of levofloxacin in wastewater.

In general, the electrocatalytic degradation of organic pollutants by anodic oxidation technology is achieved with two main reaction mechanisms: (i) oxidation of organic pollutants by direct electron transfer on the anode surface and (ii) indirect oxidation of pollutants by oxidants, such as •OH radicals produced by water electrolysis on the surface of the anode (Equation (7)), or by the generation of activated chlorine in the solution [47,62,63,64]. This study investigates the effect of the presence of chloride salts on the electrocatalytic degradation of organic pollutants. Surprisingly, the levofloxacin degradation efficiency of the titanium suboxide anode in the presence of chloride ions rapidly reached about 100% in 30 min. Chlorine ions are electrically generated in solution as Cl_2_ (Equations (8) and (9)), which further undergoes hydrolysis to form hypochlorous acid (HOCl) and hypochlorite ions (OCl^−^) (Equations (10) and (11)) [61,65,66]. The evaluation results showed that the mineralization rate of levofloxacin decreased with the increase in chloride ions (Figure 7). Although active chlorine can accelerate the degradation rate of levofloxacin, active chlorine often reacts with contaminants by unsaturated bond addition or hydrogen replacement, resulting in chlorine byproducts that are more difficult to degrade. Meanwhile, the generation of hydroxyl radicals in the process decreases with the decrease in active sites, which is also an important reason for the decrease in activity. The degradation mechanism of levofloxacin was further revealed by LC-MS characterization. As shown in Figure S6, there may be five degradation paths for LVX according to the characterization results. Among them, the pathway IV reaction process results from the attack of N-methylpiperazine groups by reactive free radicals (•OH) and reactive chlorine. The reaction via the processes of decarboxylation, defluorination, and hydroxylation continuously break down the intermediate products into small-molecule organic acids and further mineralization.

(7) $${Ti}_{n}O_{2n-1}+H_{2}O\to {Ti}_{n}O_{2n-1}\left(•OH\right)+H^{+}+e^{-}$$

(8) $${Ti}_{n}O_{2n-1}\left(•OH\right)+{Cl}^{-}\to {Ti}_{n}O_{2n-1}+1/2{Cl}_{2}(g)+{OH}^{-}$$

(9) $$2{Cl}^{-}\to {Cl}_{2}↑+2e^{-}$$

(10) $$2{Cl}_{2}\left(aq\right)+H_{2}O\to HClO+{Cl}^{-}+H^{+}$$

(11) $$HClO\to {ClO}^{-}+H^{+}$$

## 3. Materials and Methods

### 3.1. Chemicals

Levofloxacin (98.0%) was purchased from Shanghai Aladdin Biochemical Technology Co., Ltd. (Shanghai, China). Concentrated sulfuric acid (99.8%) was purchased from Sinopsin Group Chemical Reagent Co., Ltd. (Shanghai, China). Sodium sulfate (99.0%) was purchased from Shanghai Meryl Chemical Technology Co., Ltd. (Shanghai, China). Sodium hydroxide (96.0%) was purchased from Tianjin Damao Chemical Reagent Factory (Tianjin, China). Sodium chloride (99.5%) was purchased from Sinopod Group Chemical Reagent Co., Ltd. (Shanghai, China). 5,5-dimethyl-1-pyrroline-N-oxide (DMPO) was obtained from Sigma-Aldrich (St. Louis, MO, USA). Commercial TiO_2_ (analytically pure) was purchased from Sinopharm Chemical Reagent Co., Ltd. (Shanghai, China). All the chemicals and reagents were not further purified. Ultrapure water was used in the experiment.

### 3.2. Electrode Preparation

The ruthenium–titanium electrodes were purchased from Baoji Aike Metals (Baoji, China). The titanium nitrous oxide electrode preparation method was as follows: TiO_2_ was deposited on an 80 mm titanium plate by the plasma-enhanced chemical vapor deposition method; the power was 200 W; the system pressure was 53.2 Pa; the oxygen flow rate was 40 mL/min; the argon carrier gas flow rate was 2 mL/min; and the deposition temperature was maintained at 0 °C for 45 min. The resulting titanium plates coated with Ti_n_O_2n−1_ were reduced in a mixture of N_2_ and H_2_ gas (obtained by ammonia decomposition) at a reaction temperature of 850 °C and a gas flow rate of 1 L/min. The BDD electrode preparation method was as follows: boron-doped diamond film was prepared on Si substrate by chemical vapor deposition (CVD); the reaction temperature was 850 °C; the reaction gas was a 2 mL/min CH_4_ + 98 mL/min H_2_ + 0.2 mL/min B_2_H_6_ gas mixture; the gas pressure was 3 KPa; and the reaction time was 720 min.

### 3.3. Experimental Instruments

DC stabilized power supply (HY3005MT, Hangzhou Huayi Electronics Industry Co., Ltd., Hangzhou, China); fluorescence spectrophotometer (F4700, Hitachi, Tokyo, Japan); total organic carbon analyzer (TOC-L, Shimadzu (Suzhou) Instruments Manufacturing Co., Ltd., Suzhou, China); ultrasonic cleaning machine (SB-25-12DT, Ningbo Xinzhi Biological Technology Co., Ltd., Ningbo, China); tube furnace (SK2-4-12, Tianjin Zhonghuan Experimental Electric Furnace Co., Ltd., Tianjin, China); and syringe filter (25-mm diameter, 0.45-µm pore size, membrane material of polyethersulfone (PES), Tianjin Jinteng Experimental Equipment Co., Ltd., Tianjin, China).

### 3.4. Material Characterizations

The changes in the morphology of the electrodes were characterized by scanning electron microscopy (SEM) using a ZEISS Gemini 300 manufactured by Carl Zeiss (Shanghai, China) Management Co. The changes in the crystal structure of the electrodes were characterized by X-ray diffraction (XRD) using a fully automated X-ray diffractometer (Ultima IV) from Rigaku, Tokyo, Japan. Electron paramagnetic resonance (EPR) characterization of the active species in the test mechanism was performed using a Bruker A300 electron paramagnetic resonance spectrometer from Bruker, Mannheim, Germany, with the following parameters: center field: 3510 G, microwave frequency: 9.87 GHz, power: 18.11 mW, modulation frequency: 100 GHz, scanning width: 100 G, and temperature: room temperature (25 °C). The electrochemical properties of the electrodes were probed for the linear scanning voltammetry (LSV) curve. The instrument used was an electrochemical workstation model CHI760E manufactured by Shanghai Chenhua Instrument Co. (Shanghai, China).

### 3.5. Electrocatalytic Degradation of Levofloxacin

The volume of the model wastewater used for the electrocatalytic degradation of levofloxacin was 200 mL, in which the concentration of levofloxacin was 100 mg/L (Figure S1). The model wastewater was circulated into the reactor at a rate of 50 mL/min. The electrocatalytic reaction used a BDD electrode, a titanium suboxide electrode, and a Ru-Ti electrode as the anode and a Ru-Ti electrode as the cathode for the degradation reaction. And the reaction solution in the reactor was stirred by a magnetic stirrer at a speed of 300 r/min. The wastewater was sampled after the reaction to measure the concentration of levofloxacin and the concentration of TOC. The concentration of levofloxacin in the evaluation experiment was determined by high-performance liquid chromatography (HPLC, P1201). In order to ensure that the reaction proceeded properly, 3‰ of Na_2_SO_4_ was added to each group of reactions (Na_2_SO_4_ acts as a supporting electrolyte in the reaction). The degradation process applied a current density of 39.6 A/m^2^.

### 3.6. Analytical Methods

The degree of mineralization of the characteristic pollutant levofloxacin is indicated by the change in total organic carbon (TOC), which was tested by the TOC analyzer (TOC-L CPN, Shimadzu, Kyoto City, Japan).

(12) $$TOC\;removal=\frac{TOC_{0}-{TOC}_{t}}{{TOC}_{0}}\times 100\%$$

where TOC_0_ is the initial total organic carbon of the wastewater, and TOC_t_ is the total organic carbon of the wastewater after the reaction.

(13) $$Levofloxacin\;Conversion=\frac{C_{0}-C_{t}}{C_{0}}\times 100\%$$

where C_0_ is the initial concentration of levofloxacin, and C_t_ is the concentration of levofloxacin after the reaction.

## 4. Conclusions

In summary, this paper discussed the effect of sodium chloride addition on the efficiency of three anodic electrodes used for the electrocatalytic degradation of levofloxacin wastewater. By comparing the performances of the three electrodes, it was determined that chloride ions improved the efficiency of the electrocatalytic degradation of levofloxacin by the titanium suboxide and Ru-Ti electrodes. Through the evaluation experiments and EPR characterization, it was found that hydroxyl radicals and active chlorine played a major contributing role in the degradation process. The electrochemical performance test showed that the concentration of chloride ions affected the oxygen precipitation potential of the electrode and thus affected the formation of hydroxyl radicals. The study reveals the mechanism of chloride ions involved in the reaction during the electrocatalytic degradation of chlorinated organic wastewater. In addition, this study also provides theoretical references for the development of technologies for treating saline organic wastewater.

## Data Availability

Data are contained within the article and Supplementary Materials.

## Supplementary Materials

The following supporting information can be downloaded at: https://www.mdpi.com/1420-3049/29/3/662/s1, Figure S1: Diagram of electrode electrooxidation device; Figure S2: (a) XRD patterns and (b) SEM images of BDD; Figure S3: EPR signal of hydroxyl radical in the degradation of levofloxacin by titanium oxide and Ru-Ti electrode: (a) Ti_4_O_7_ without NaCl; (b) Ti_4_O_7_ + NaCl; (c) Ru-Ti without NaCl; (d) Ru-Ti + NaCl; Figure S4: Effect of tert-butanol (TBA) on the degradation of levofloxacin by BDD, titanium suboxide and Ru-Ti electrodes (a) LVX conversion and (b) TOC removal; Figure S5: Effect of pH for degradation Levofloxacin; Figure S6: Proposed degradation pathways of LVX during electrooxidation reaction; Table S1: Comparison of overpotentials of titanium suboxide and Ru-Ti electrodes at different chloride concentrations.
