# Supplementary material for: Boosted Electrocatalytic Degradation of Levofloxacin by Chloride Ions: Performances Evaluation and Mechanism Insight with Different Anodes
Source: Molecules. 2024 Jan 31;29(3):662. doi: 10.3390/molecules29030662 (PMC11487383; doi:10.3390/molecules29030662)
Supplement: Supplementary file 1 [file molecules-29-00662-s001.zip › molecules-2841212-supplementary.pdf]

Boosted electrocatalytic degradation of levofloxacin by chloride ions: performances evaluation and mechanism insight with different anodes

Keda Yang[1&]， Peiwei Han[2&], Yinan Liu[2], Hongxia Lv[2], Xiaofei Chen[3], Yihan Lei[3], Lian Yu[4], Lei Ma[2*], Pingzhou Duan[5*]

[1]Shulan International Medical College, Zhejiang Shuren University, Hangzhou 310015, China;

[2]Beijing Key Laboratory of Fuels Cleaning and Advanced Catalytic Emission Reduction Technology, College of New Materials and Chemical Engineering, Beijing Institute of Petrochemical Technology, Beijing 102617, China;

[3]Chen Ping Laboratory of TIANS Engineering Technology Group Co., Ltd., Shijiazhuang 050000, Hebei, China;

[4]Department of Environmental Engineering, Beijing Institute of Petrochemical Technology, Beijing 102617, PR China

[5]State Key Laboratory of Environmental Criteria and Risk Assessment, Chinese Research Academy of Environmental Sciences, Beijing 100012, China;

[&]These authors contributed equally.

[*] **Correspondence:**
E-mail: malei@bipt.edu.cn

E-mail: duanpz@craes.org.cn

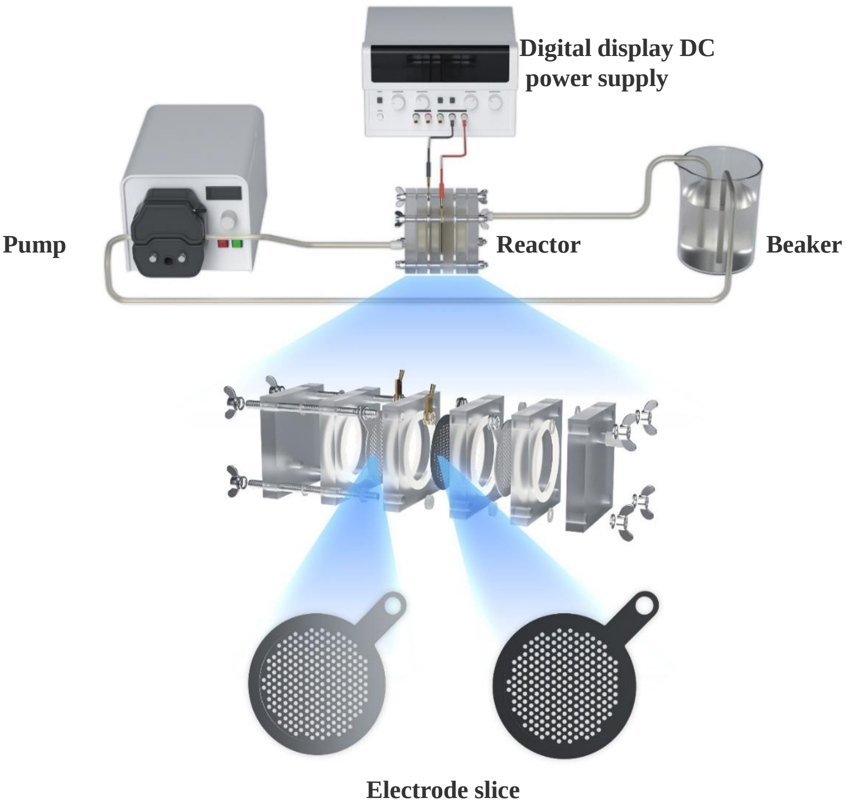

Figure S1 Diagram of electrode electrooxidation device

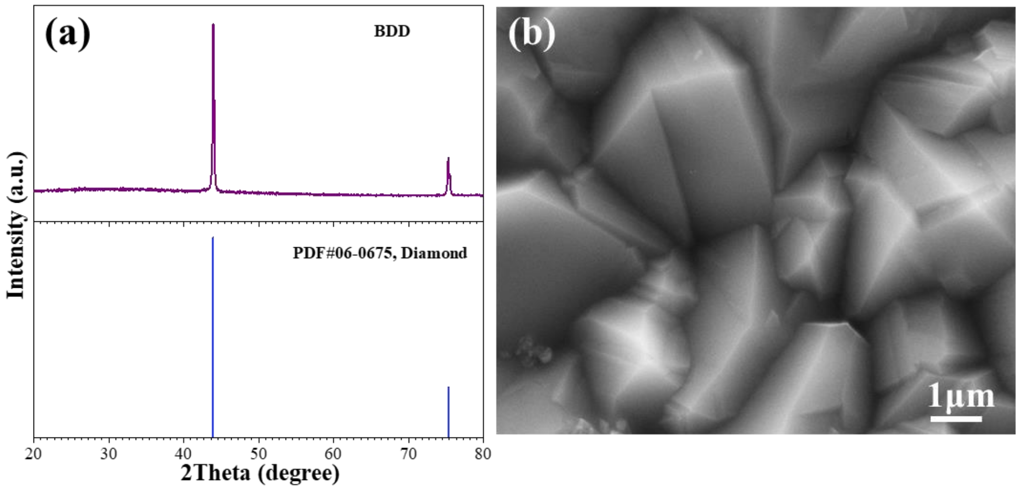

Figure S2 (a) XRD patterns and (b) SEM images of BDD

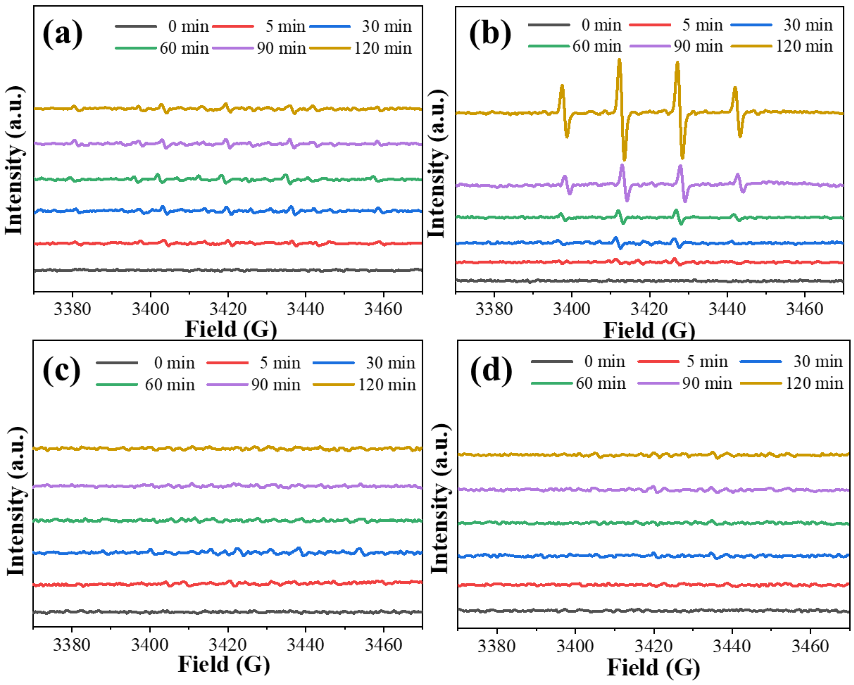

Figure S3 EPR signal of hydroxyl radical in the degradation of levofloxacin by titanium oxide and Ru-Ti electrode: (a) Ti_4O_7 without NaCl; (b) Ti_4O_7 + NaCl; (c) Ru-Ti without NaCl; (d) Ru-Ti + NaCl.

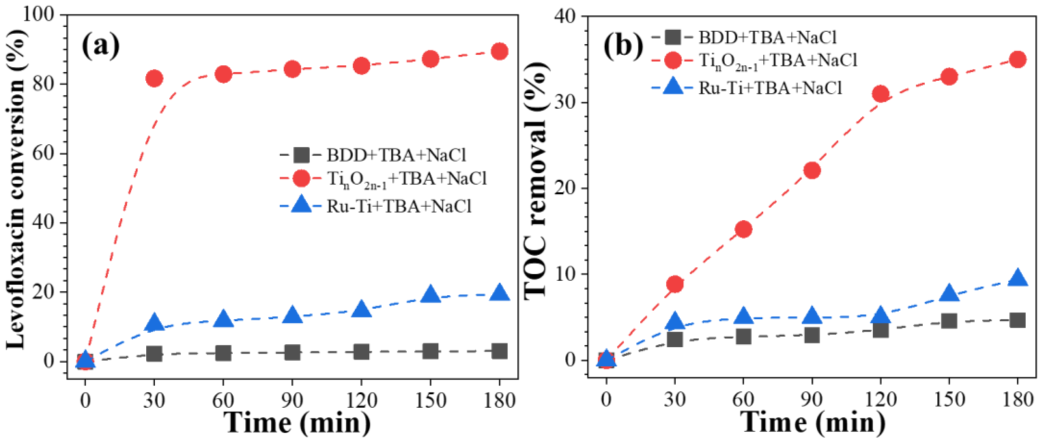

Figure S4 Effect of tert-butanol (TBA) on the degradation of levofloxacin by BDD, titanium suboxide and Ru-Ti electrodes (a) LVX conversion and (b) TOC removal.

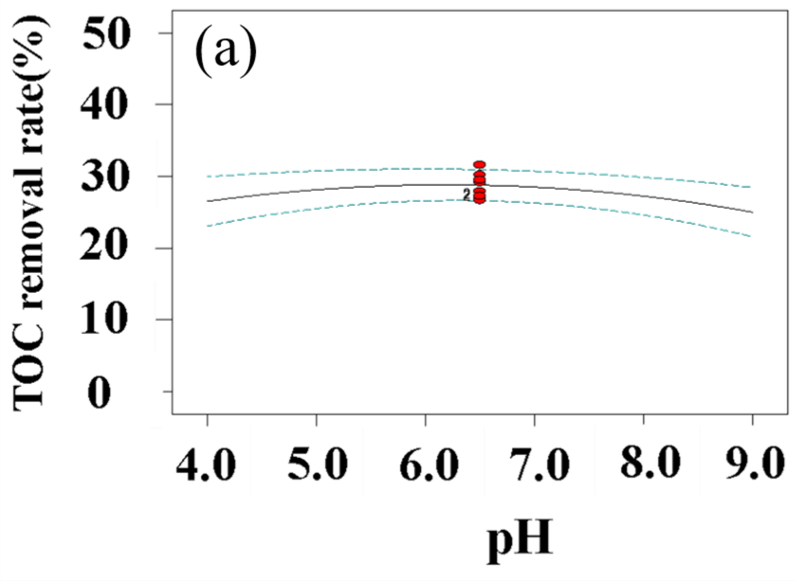

Figure S5 Effect of pH for degradation Levofloxacin

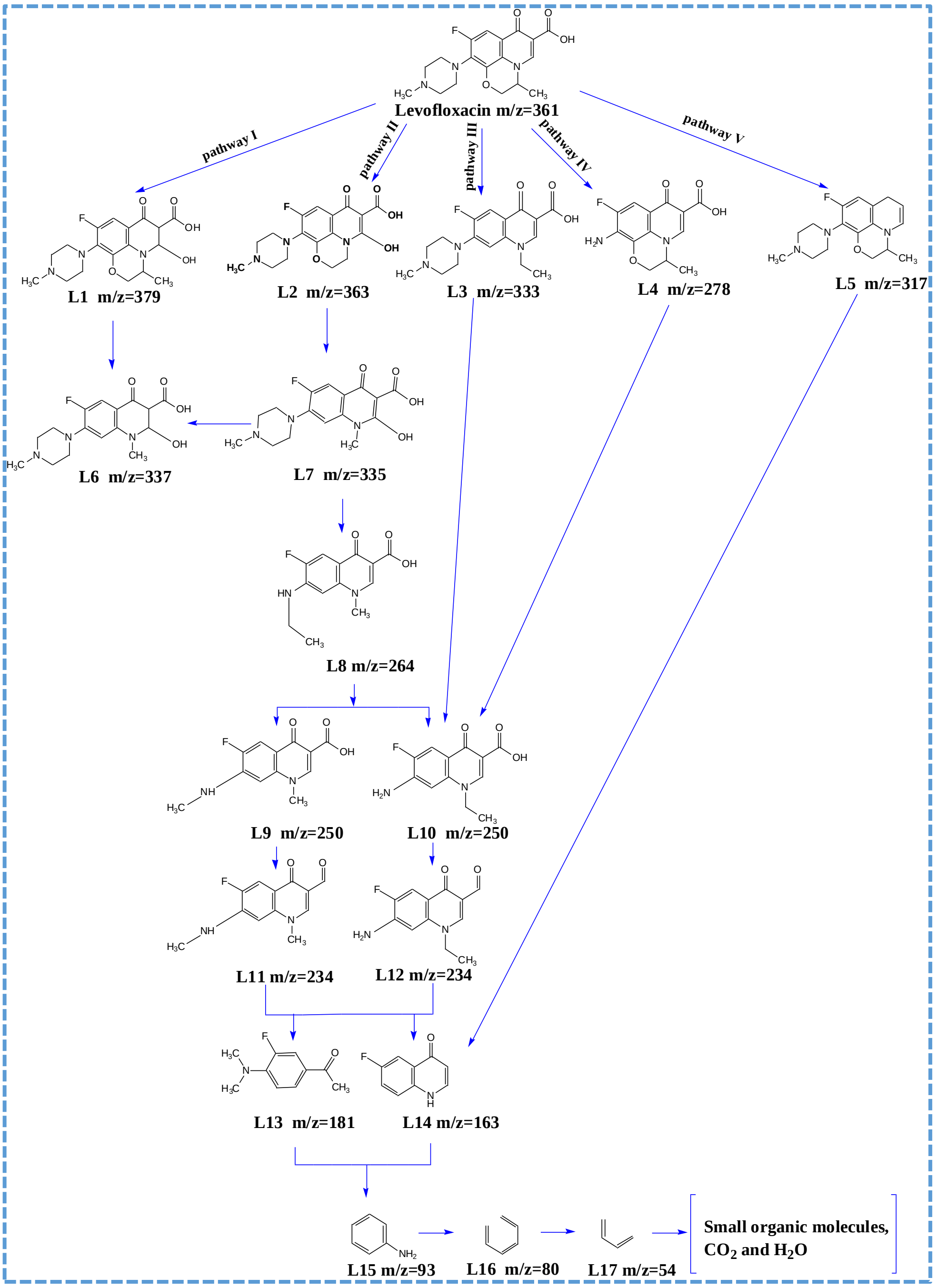

Figure S6 Proposed degradation pathways of LVX during electrooxidation reaction

Table S1. Comparison of overpotentials of titanium suboxide and Ru-Ti electrodes at different chloride concentrations

samples	Over potential (V vs. SCE)
**Ti_nO_{2n-1}-0‰ Cl^-**	1.95
**Ti_nO_{2n-1}-4‰ Cl^-**	2.08
**Ti_nO_{2n-1}-8‰ Cl^-**	1.98
**Ti_nO_{2n-1}-12‰ Cl^-**	1.88
**Ru-Ti-0‰Cl^-**	1.30
**Ru-Ti-4‰Cl^-**	1.23
**Ru-Ti-8‰Cl^-**	1.18
**Ru-Ti-12‰Cl^-**	1.15
